# Reversing Coffee-Ring Effect by Laser-Induced Differential Evaporation

**DOI:** 10.1038/s41598-018-20581-0

**Published:** 2018-02-16

**Authors:** Tony M. Yen, Xin Fu, Tao Wei, Roshan U. Nayak, Yuesong Shi, Yu-Hwa Lo

**Affiliations:** 1 Department of Bioengineering, University of California San Diego, La Jolla, CA 92093-0412 United States; 2 Chemical Engineering Program, University of California San Diego, La Jolla, California 92093-0448 United States; 3 Department of Chemical Engineering, Howard University, Washington, DC 20059 United States; 4 Materials Science Program, University of California San Diego, La Jolla, California 92093-0418 United States; 5 Department of Electrical and Computer Engineering, University of California San Diego, La Jolla, California 92093-0407 United States

## Abstract

The coffee-ring effect, ubiquitously present in the drying process of aqueous droplets, impedes the performance of a myriad of applications involving precipitation of particle suspensions in evaporating liquids on solid surfaces, such as liquid biopsy combinational analysis, microarray fabrication, and ink-jet printing, to name a few. We invented the methodology of laser-induced differential evaporation to remove the coffee-ring effect. Without any additives to the liquid or any morphology modifications of the solid surface the liquid rests on, we have eliminated the coffee-ring effect by engineering the liquid evaporation profile with a CO_2_ laser irradiating the apex of the droplets. The method of laser-induced differential evaporation transitions particle deposition patterns from coffee-ring patterns to central-peak patterns, bringing all particles (e.g. fluorescent double strand DNAs) in the droplet to a designated area of 100 μm diameter without leaving any stains outside. The technique also moves the drying process from the constant contact radius (CCR) mode to the constant contact angle (CCA) mode. Physical mechanisms of this method were experimentally studied by internal flow tracking and surface evaporation flux mapping, and theoretically investigated by development of an analytical model.

## Introduction

The drying of a droplet of water carrying colloidal particles naturally gives rise to non-homogenous deposition pattern, with most of particles migrating to the edge of the droplet, forming the well-known coffee-ring deposition pattern^1^. This non-uniform deposition has posed technical challenges in inkjet printing^2^, DNA/RNA and protein microarray manufacturing^3,4^, and most recently in combinational liquid biopsy analysis methods such as fluorescent microarray, infrared spectroscopy, and Raman spectroscopy^5,6^. While modification of surfactants additives^4^, colloidal geometric shape^7^, salt concentration, and colloidal particle size^8^ in aqueous solution have been shown to be effective in generating homogenous depositions, controllability of pattern sizes from these methods remains an issue. In most cases, the size of the uniformed deposition patterns duplicates the size of coffee-ring patterns and cannot be reduced. To increase the quality in printing, microarray fabrication and analysis, and in advancing liquid biopsy analysis sensitivity and accuracy, substantial reduction in the pattern size is desired.

As described in the seminal work by Deegan *et al*.^1^, the physical cause of the coffee-ring effect is an outward, radial flow initiated by a weakly pinned contact line and maximum evaporation flux at the edge of the droplet. Thermally-induced Marangoni effect has been proven effective to create a re-circulation flow to reverse the coffee-ring effect, and to reduce the deposition pattern size of solution droplets with volatile solvents^9,10^. However, when applied onto aqueous droplets, the thermally-induced re-circulation flow is suppressed and the coffee-ring effect dominates the Marangoni effect^9^. Electro-wetting has also been shown effective to reduce the deposition pattern size of drying aqueous droplet, but requires relatively high concentration (10 mM) of LiCl additive to adjust the solution’s conductivity^11^. To recover signals of diluted (100 nM to 100 aM) analyte from the dried LiCl solid crystal poses yet another technical challenge. To fundamentally improve aqueous sample analysis from a drying droplet, the following criteria must be satisfied: reversing of coffee-ring effect, reduction in deposition pattern size, and minimal or preferably no additive to the solution.

Besides experimental studies, the coffee-ring phenomenon has also been examined both computationally and analytically^12^. The complex interplay between hydrodynamic effect^13^, thermal effect^14^, evaporative conditions^15^, and colloidal particle concentration and surface adsorption^16^ has triggered great difficulties in producing multi-variate physical insights from pure computational modeling. Most computational modeling studies focus on few effects in isolation with limited physical conditions^17^. Analytical approaches are much more versatile in describing the complex interplays of different effects involving moving contact line^12^. The seminal work by Man and Doi^18^ has provided great physical insights into the droplet drying pattern based on analytical solutions.

Inspired by the extensive studies on the coffee-ring effect, a central idea was developed that the coffee-ring effect can be removed effectively provided there is a method to produce an environment that the evaporation rate at the apex of the droplet is much faster than the evaporation rate on surfaces elsewhere. The strong evaporation rate at the central region of the droplet will provide the needed driving force for the Marangoni flow that suppresses the coffee-ring effect. Given the small size of the droplets in most applications, we propose the idea of using a CO_2_ laser to create the effect of laser-induced differential evaporation. Water molecules have strong absorption (greater than 3000 cm^−1^) at 10.6 μm wavelength of CO_2_ laser, hence is highly effective in generating a differential evaporative flux profile on water surface. This laser-induced differential evaporation method has successfully reduced the deposition pattern size of aqueous solution droplets from 1.5 mm to the designated 100 μm spot without addition of any ionic salts or surfactants. Moreover, our setup is low-power (40 mW) and can be scaled up with parallel optical paths in an array format. During droplet evaporation, the CO_2_ laser beam creates maximum evaporative flux at the apex of the droplet, creating an inward, radial flow that dominates and counteracts the outward, radial flow of coffee-ring effect. The inward flow occurs concurrently with contact line de-pinning and ultimately leads to the peak deposition patterns. Substantiating our experimental data and qualitative arguments, approximate analytical solutions were derived based on Man and Doi’s original analysis of drying patterns^18^. Jointly with the experimental results, the analytical solutions derived in this paper confirm the importance of differential evaporation in reversing the coffee-ring effect.

## Results

### CO_2_ laser system setup and characterization

**Figure 1:**
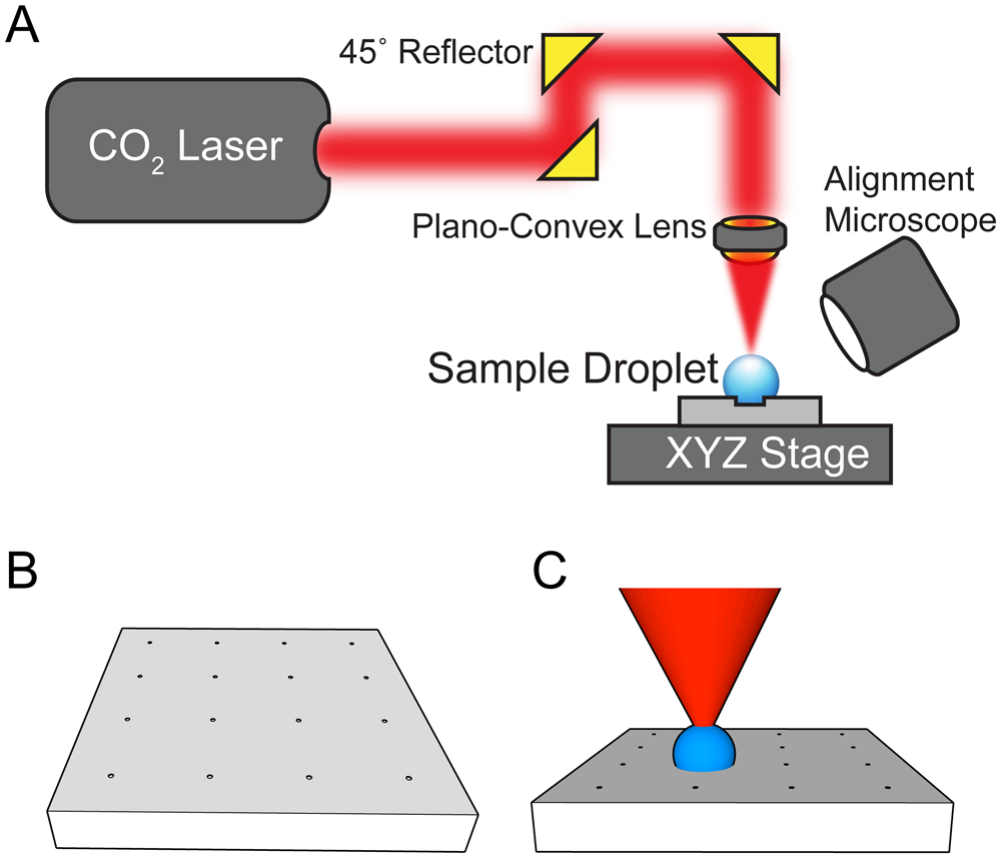
CO_2_ laser system for differential evaporation. (**A**) The carbon dioxide laser beam is collimated through a beam expander (not shown here), reflected at a 45° angle 3 times, and attenuated 75% to achieve top-down exposure of the plano-convex lens. Through the plano-convex lens, the laser beam converges into a focal point. Using an alignment microscope and a XYZ linear stage, a sample droplet is centered to the laser beam’s focal point and a high evaporation flux occurs at the apex of the droplet. (**B**) Cytop coated microarray with 4 × 4 100-µm diameter well patterns with 2 mm spacing. (**C**) Microarray architecture allows the laser system to continuously deposit biomolecules from solution droplets on the XY plane.

We have designed our CO_2_ laser system to generate maximal evaporation flux difference on solution droplet surface. To realize such effect, the CO_2_ laser beam was focused to a 29 µm spot on the glass substrate, which is the basal surface of the droplets. The optomechanical and imaging setup is shown in Fig. 1A. The light source is a 10 W CO_2_ laser (Universal Laser Systems ULR-10) with emission wavelength of 10.6 μm, beam size of 4 mm, and divergence of 5 mrad. The laser power is modulated at a frequency of 20 kHz and duty cycle ranging from 0–100%. To achieve the desired size of beam spot, the laser beam enters a 10x beam expander composed of a pair of plano-concave (focal length−50 mm, diameter 1″) and plano-convex (focal length 500 mm, diameter 1″) zinc selenide (ZeSe) lenses with broadband (7–12 μm) antireflective coating. The expanded laser beam with diameter of 40 mm is reflected three times at a 45° angle using gold-coated mirrors to allow the beam direction to change from parallel to perpendicular to the sample stage. Since differential evaporation requires much lower laser power (40 mW), a piece of 22 mm glass circular coverslip is fixed to the center of one of the gold-coated mirrors to achieve 75% attenuation. Lastly the laser beam passes through a plano-convex (focal length 50 mm, diameter 1″) ZeSe lens and converges into a focal point. The position of the laser focal point is recorded using an alignment microscope (Dino-Lite AM411T) mounted at a 30–40° angle from the sample stage.

To define the sites for sample enrichment and/or reactions (hybridization or binding) in an array format, the glass substrate is coated with amorphous fluorocarbon polymer Cytop with 100 μm-diameter SiO_2_ wells. Since Cytop-coated surface is hydrophobic (100° in contact angle, 4.5 μm in thickness), the entire Cytop microarray device surface is hydrophobic except the uncovered, 100 μm-diameter SiO_2_ wells (43° in contact angle), as shown in Fig. 1B. The Cytop coating encourages water droplet to de-pin from its surfaces while the hydrophilic well anchors droplet onto the pattern throughout the drying process. Each Cytop microarray device is 8 mm by 8 mm in dimension and has 4 × 4 100-μm diameter well patterns spaced 2 mm apart (See Methods for Cytop microarray fabrication). During experimental operation, sample droplets are spotted onto patterns using a syringe-pump droplet spotting system (See Methods for droplet spotting system setup). To start the differential evaporation process, the laser is turned on at 40 mW and the laser beam is focused onto the spotted droplet (Fig. 1C). As the sample droplet dries, the laser power is reduced sequentially to be approximately proportional to the 3^rd^ power of the receding radius of the droplet (as suggested by the theoretical analysis discussed later).

### Effect of laser-induced differential evaporation on biomolecule deposition

**Figure 2:**
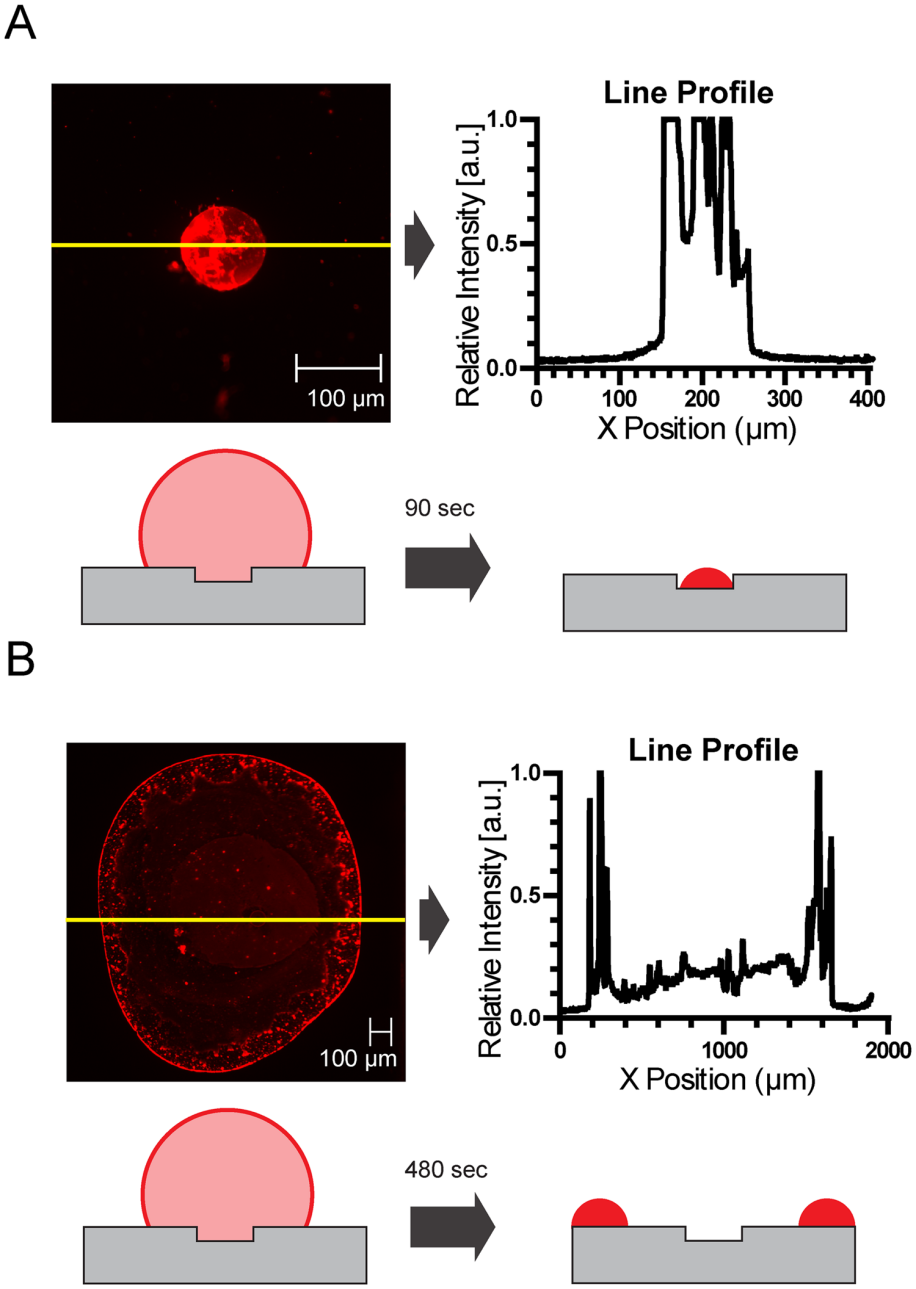
Image and line profile comparison between fluorescent DNA molecules deposited on Cytop microarray by laser heating and hot plate heating. The deposited fluorescent DNA molecules are single-stranded, FAM labeled, and 24 base pairs in length. (**A**) Differential evaporation by laser heating results in DNA molecule deposition patterns confined within the 100 µm patterns. The average time for 1 µL of 10nM solution droplet to dry is ~90 s. (**B**) Hot pate heating results in a typical coffee-ring pattern, in which the DNA molecule dries in a ring structure (~1.5 mm) outside of the 100 µm patterns. The average time for 1 µL of 10nM solution droplet to dry is ~480 s for 50 °C hot plate.

To demonstrate the effect of laser-induced differential evaporation on biomolecule deposition, we have dried 1 µL droplets that contain 10 nM fluorescent ssDNA (single stranded FAM labeled DNA, 24 nts in length) using both laser-induced differential evaporation method and a hot plate setup. The hot plate method accelerated solution droplet evaporation by heating up droplets from the bottom liquid-solid contact. Fig. 2 shows fluorescent DNA deposition patterns on Cytop microarray device resulting from both the laser-induced method and the hot plate heating (50 °C). The deposition patterns were imaged using an enclosed fluorescent microscope (See Methods for deposition pattern imaging and analysis). For the differential evaporation method, fluorescent image and line profile analysis shows insignificant fluorescent signal outside of the 100 μm microarray pattern (Fig. 2A); suggesting minimal liquid pinning event during drying and absence of droplet edge-deposition. For the hot plate method, fluorescent image and line profile analysis show prominent fluorescent signal in a ring-structure with diameter ~1.5 mm, suggesting significant liquid pinning event during drying and droplet edge-deposition, the so-called coffee-ring effect (Fig. 2B). Using ssDNA of 24 base pairs in our experiments, we observed a smooth triple line (TL) DNA deposition for hot plate heating method under optical microscope (Fig. 2B) instead of dendrite crystal formation. Our observation is in agreement with the previous report of DNA orientation effect on TL deposition for shorter nucleotide (100 bp) from Askounis *et al*.^19^. In terms of overall evaporation rate, the differential evaporation method requires ~90 sec to dry 1 µL of the 10 nM solution droplet, and the hot plate heating requires ~480 sec for complete evaporation. With a 16 × 16 commercially available ZnSe microlens array with 1.5 mm pitch, our method can be extended to dry 256 droplets simultaneously with the compact CO_2_ laser.

Despite sharing identical fluidic properties, bottom substrate surface material and geometry, and ambient environment conditions with the hot plate heating method, the differential evaporation method has avoided coffee-ring deposition in a repeatable fashion. Table 1 shows repeating results (n = 6) of deposition pattern size and capture ratio from both differential evaporation and hot plate methods (1 µL of 10 nM fluorescent ssDNA). Pattern size quantifies the spread of the deposition pattern, and capture ratio measures the relative amount of fluorescent signal detected in the 100 μm pattern region compared to the total signal of the imaged surface (See Methods for deposition pattern imaging and analysis). For the differential evaporation method, repeating deposition testing yields an average drying pattern size of 101 μm, size standard deviation of 1 μm, and an average capture ratio of 73.2%, indicating that most fluorescent DNA molecules were deposited within the pattern. The capture ratio is less than 100% possibly due to either device background auto-fluorescence, background light scattering, or non-specific DNA surface adsorption. Nevertheless, in the case of coffee-ring deposition by hot plate heating, the average capture ratio is 0.306%. A low capture ratio, combined with an average pattern size of 1504 μm, indicates that most fluorescent DNA are preferentially deposited outside of the microarray 100 μm pattern. Both qualitative and quantitative analysis indicates differential evaporation causes significant shift in the mode of deposition from the typical coffee-ring deposition.

**Table 1:** Deposition pattern size and capture ratio vs. drying method.

Method:	Differential Evaporation		Hot Plate	
n	Pattern Size (μm)	Capture Ratio (%)	Pattern Size (μm)	Capture Ratio (%)
1	102	75.6	1435	0.308
2	102	70.3	1736	0.228
3	101	82.3	1456	0.316
4	100	73.3	1292	0.330
5	101	68.1	1571	0.325
6	99	69.6	1535	0.330
Average	101	73.2	1504	0.306
Standard Deviation	1	5.2	149	0.039

### Effect of differential evaporation on mode of droplet evaporation

**Figure 3:**
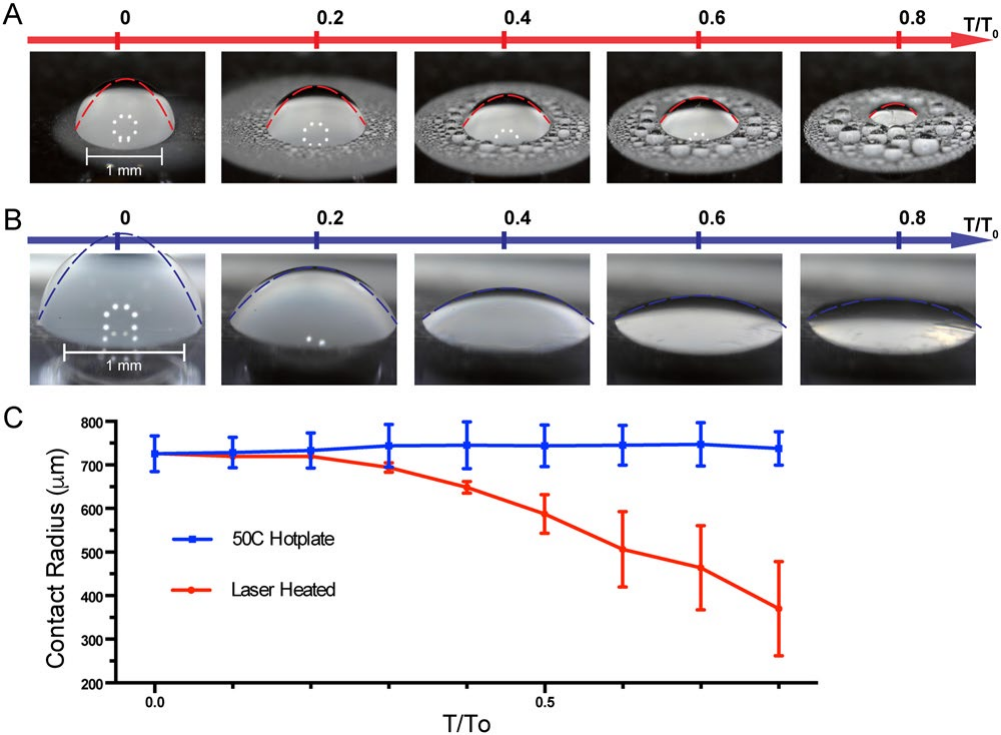
Modes of droplet evaporation from differential evaporation and hot plate heating on Cytop microarray. (**A**) 1 µL of 10 nM ssDNA droplet undergoes differential evaporation. The droplet shape is fitted to parabolic curve in red dashed line. As time progresses, the droplet volume and contact radius shrank due to water evaporation at droplet apex. Micro-droplets condense around the heated droplet due to water vapor diffusion from active evaporation and temperature gradient of the relatively cooled bottom surface. (**B**) 1 µL of 10nM ssDNA droplet is heated by 50 °C hot plate. The droplet shapes are fitted to parabolic curves in blue dashed line. As time progresses, the droplet volume shrinks due to water evaporation from bottom hot plate heating. No surface condensation is observed since the solid surface is higher in temperature. (**C**) Contact radius time-evolution curves. Contact radius for differentially evaporated droplet continues to shrink with time, while hot plate dried droplet is pinned at its original position.

To establish the physical connection between differential evaporation and the mode of biomolecule deposition, we investigated the mode of droplet evaporation during differential evaporation. The mode of droplet evaporation characterizes the evolution history of droplet contact line position and contact angle. While there are two pure droplet evaporation modes, one with constant contact radius (CCR) and the other one with constant contact area (CCA), most aqueous droplet evaporation undergoes both pure modes as well as a mixture of two modes where both contact area and contact radius are variable^20,21^. The transition from CCA to CCR mode is intrinsically related to pinning of the triple phase contact line. Fig. 3 shows the droplet evaporation mode during both differential evaporation and hot plate heating (50 °C) on Cytop microarray device (1 µL of 10 nM fluorescent ssDNA). Since the overall rate of evaporation is different between the two methods, progressions are normalized to its relative time, T/T_0_, where T_0_ is the time for complete evaporation (~90 sec for differential evaporation and ~480 sec for hot plate heating). Contact radius data are calculated from analysis of tilted angle (30–40° from parallel) video recordings to allow real time observation during evaporation. In the case of differential evaporation, water condensation occurs due to oversaturation of water vapor in surrounding areas of the droplet. We stop the contact radius measurements when water condensation occludes the view and affects measurement accuracy (at around T/T_o_ = 0.8).

Droplet evaporation recordings reveals 2 key differences between the two evaporation processes. One key difference is the surface condensation on the Cytop microarray during differential evaporation, forming micro-droplets around the original droplet, while hot plate heating is free of surface condensation (Fig. 3A). In the hot plate heating setup, the device surface temperature is elevated to 50 °C relative to the ambient temperature (~20 °C) and thus water vapor rapidly leaves the surface and diffuses into the ambient (Fig. 3B). On the other hand, during differential evaporation, the substrate surface is at ambient temperature, thus is in favor of condensation of the hot vapor oversaturating the surrounding region. The other key difference is contact line movement. During differential evaporation (Fig. 3C), the droplet contact line continues to de-pin from the Cytop surface and moves toward the center, while hotplate heating results in a pinned contact line throughout droplet evaporation. The contact radius time-evolution curve shows a time-independent, constant contact radius of 750 μm for hot plate heating, matching the CCR mode of evaporation. In contrast, laser-induced differential evaporation results in a receding contact radius with time progression. During the initial stage of evaporation (T/T_0_ ~ 0.0–0.2) for differential evaporation (Fig. 3A), droplet contact angle decreases in the CCR mode of evaporation (Fig. 3C) accompanied with re-condensation at TL (snapshots of Fig. 3A) at high humidity and with large dynamic contact angle, which is similar to the previous report for a droplet in natural evaporation at the initial stage^22^. However, from T/T_0_ ~ 0.3 to the final, the droplet undergoes further evaporation with subsequent decrease in the droplet radius, mainly due to the CO_2_ laser induced heating and hydrodynamic flow effect^23^. Moreover, during the 0.4 to 0.8 T/T_0_ time regime, the normalized contact radius receding rate is linear ($i.e.\dot{R}/R \sim Ct$) (also see supplementary materials), showing the characteristic of the CCA mode of evaporation. During differential evaporation, the normalized contact angle rate ($i.e.\dot{\theta }/\theta$) declines with time and essentially approaches zero, again showing the characteristic of the CCA mode of evaporation. Comparing between contact radius and contact angle data for the two processes, a shift from CCR to CCA evaporation mode is observed for the laser-induced differential evaporation method.

### Inward, radial flow from laser-induced differential evaporation

**Figure 4:**
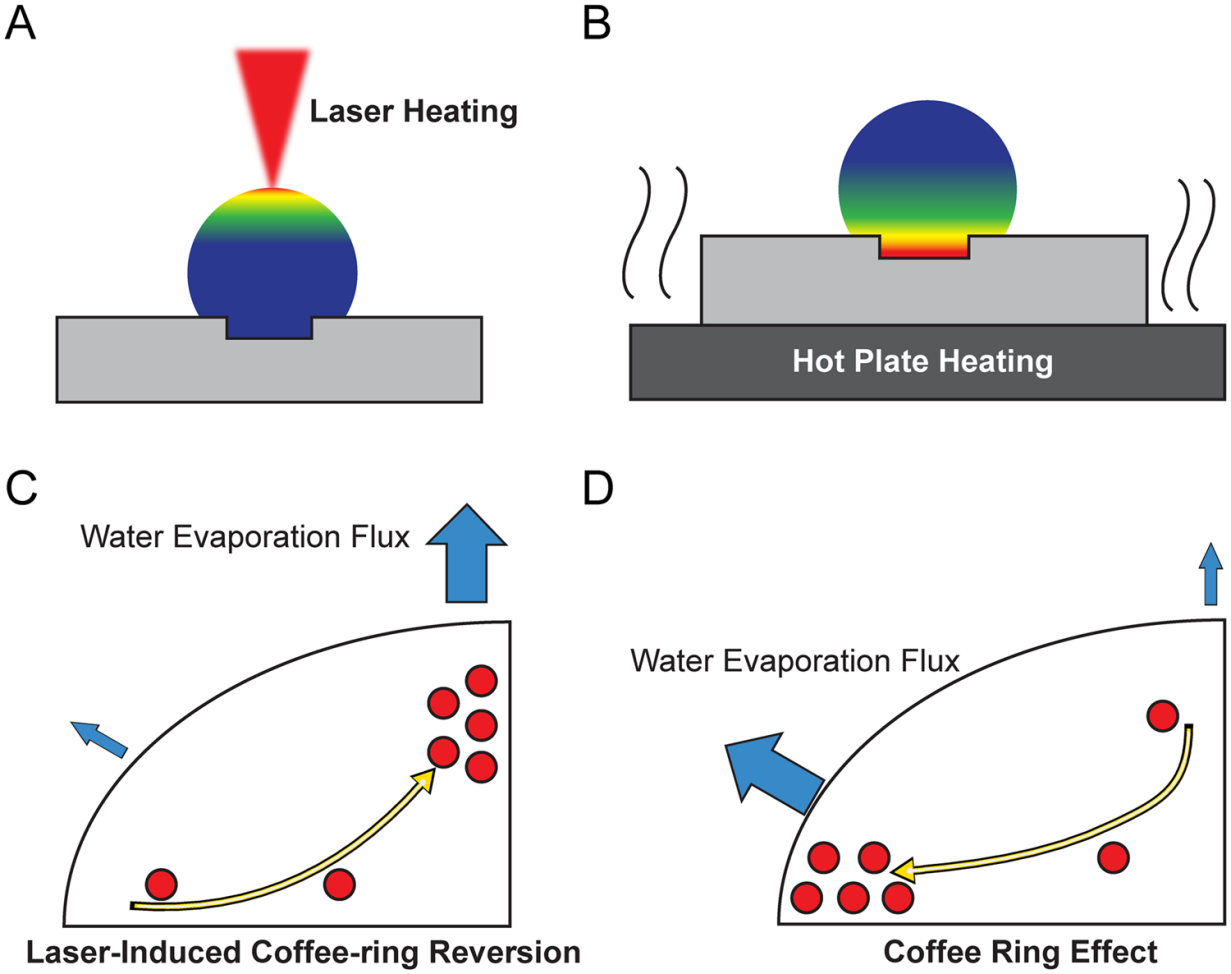
Thermal and water evaporation flux response to laser heating. (**A**) Laser heating at the droplet apex creates a temperature gradient and evaporation rate that is highest at droplet apex and lowest at droplet base. (**B**) Hot plate heating at the droplet base creates a temperature gradient that is highest at droplet base and lowest at droplet apex. (**C**) Due to the surface laser heating on the droplet apex, water evaporation flux is highest at the droplet apex. An internal flow develops to replenish the lost water volume at the apex, producing an inward, radial flow that carries colloidal particles to the droplet center and reverses the coffee-ring effect. (**D**) Due to lower probability of water vapor reabsorption at droplet edge, water evaporation flux is highest at the droplet edge. Governed by the mass transport equation, an internal flow must be supplied to replenish the water loss at the edge. As a result, an outward, radial flow carries colloidal particles to the droplet edge, causing the coffee-ring effect.

As pointed out by previous studies on colloidal liquid evaporation, the physical cause behind contact line pinning is a weakly pinned contact line and the suppression of thermally-induced Marangoni flow, which then leads to an outward, radial flow causing the coffee-ring effect^1,9^. It is noteworthy that the previous study^22^ of Shanahan and the co-workers illustrated the effect of re-condensation on the droplet natural evaporation. Our current work focuses on the localized external heating. Since the re-condensed droplets contain no particles and do not convalesce with the original droplet that is shrinking, the re-condensation effect is not included in the current model. Fig. 4 shows liquid temperature isotherm, water evaporation flux distribution, and internal fluidic flow diagrams for both laser-induced differential evaporation and hot plate heating methods. The liquid isotherm diagram shows laser induced evaporation creates a temperature profile that is highest at droplet apex and lowest at droplet base (Fig. 4A). In contrast, hotplate heating creates a temperature profile with the highest temperature at droplet base (Fig. 4B).

To understand how laser heating leads to differential evaporation at the droplet’s surface, we can describe the relation between water evaporation flux J^*^ and interface quantities via the Hertz-Knudsen expression from kinetic theory of gas^12^, ${J}^{\ast }=\alpha \sqrt{\frac{M}{2\pi \bar{R}{T}_{sat}^{\ast }}}[{p}_{sat}^{\ast }({T}_{i}^{\ast })-{p}_{v}^{\ast }]$, where ${p}_{sat}^{\ast }({T}_{i}^{\ast })$ is the saturation pressure at the interface temperature ${T}_{i}^{\ast }$, ${T}_{sat}^{\ast }$ is the saturation temperature, ${p}_{v}^{\ast }$ is the vapor pressure just beyond the interface, and $\bar{R}$ is the universal gas constant. The parameters α and M are the accommodation coefficient (measure of liquid volatility) and the molecular mass of vapor respectively. For most practical cases, the coefficients α, M, $\bar{R}$, and ${T}_{sat}^{\ast }$ can be considered nearly constant throughout the process of droplet evaporation. For laser induced differential evaporation in Fig. 4C, J^*^ reaches maximum at the droplet apex because surface temperature maximum yields maximum $[{p}_{sat}^{\ast }({T}_{i}^{\ast })-{p}_{v}^{\ast }]$. Moreover, ${p}_{v}^{\ast }$ is much lower than ${p}_{sat}^{\ast }({T}_{i}^{\ast })$ above the droplet apex because the laser beam also heats up the water vapor just beyond the droplet apex surface to drive the vapor away. The combined effect of elevated surface saturation pressure and removal of external water vapor gives rise to the laser-induced differential evaporation phenomenon. Based on the equation of mass transport, the strong water evaporation flux at the droplet apex leads to an inward, radial flow toward the droplet center, carrying colloidal particles toward the center of droplet. For the hot plate heating analysis in Fig. 4D, as explained by Deegan *et al*.^24^, local evaporation rate diverges and reaches maximum at the droplet edge because of lower probability of water vapor reabsorption at droplet edge. Therefore, an outward, radial flow develops, carrying the colloidal particles to the droplet edge to produce the coffee-ring effect.

### Surface vapor pressure and colloidal particle movement from laser-induced differential evaporation

To support the proposed physical mechanisms, the local saturation pressure and droplet internal flow direction is experimentally verified. Since there is no direct method to assess the local saturation pressure surrounding the droplet, the droplet surface temperature is recorded to extrapolate the saturation pressure at the surface. In our case, the droplet surface temperature is imaged using a microbolometer (FLIR A655sc) at a resolution of 640 × 480 pixels and at 25 μm spatial resolution. To measure the internal fluid flow, polystyrene micro-beads are added to the solution to track their motions from recorded images.

**Figure 5:**
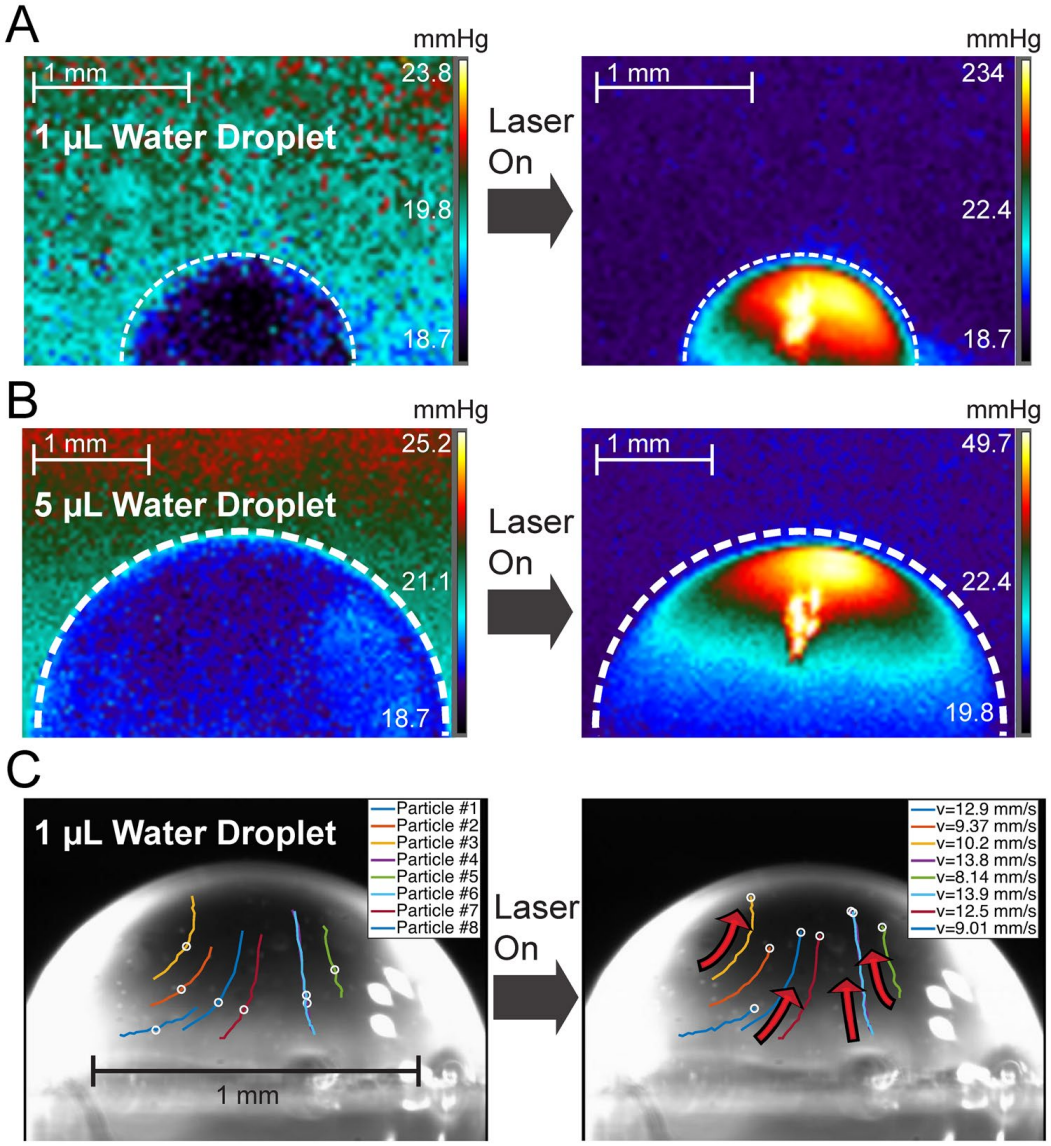
Surface water vapor saturation pressure and droplet internal flow tracking by polystyrene beads from laser-induced differential evaporation. (**A**) Laser-induced differential evaporation (40 mW) on 1 µL water droplet yields a saturation pressure of 234 mmHg on the droplet apex surface. (**B**) Laser-induced differential evaporation (40 mW) on a 5 µL droplet yields a saturation pressure of 49.7 mmHg on the droplet apex surface. Oversaturated spots in (**A**) and (**B**) are artifacts caused by reflections of the IR laser. (**C**) Polystyrene micro-beads (3.3 μm in diameter, 800 beads/μL in water) movement tracking during a 33.3 ms timeframe shows upward, centripetal fluidic flow with a mean velocity of 11.2 mm/s toward the droplet apex in the 1 µL droplet.

Figure 5 shows surface water vapor saturation pressure and polystyrene bead movement during laser-induced differential evaporation. Two volumes of water droplet (5 μL and 1 μL) are imaged to demonstrate the direct effect of laser beam size on surface water vapor saturation pressure (Fig. 5A,B). The laser beam diameter is ~100 μm and ~60 μm respectively on 5 μL (~2 mm of liquid height) and 1 μL (~0.85 mm of liquid height) water droplet apex. Under the same laser power of 40 mW, the 100 μm and 60 μm laser beam diameters correspond to saturation pressure of 234 mmHg and 49.7 mmHg on droplet apex. This observation correlates to different progression during the differential evaporation process. As the droplet size reduces, surface saturation pressure increases at droplet apex. In addition, the corresponding surface temperature recorded at the droplet apex is 70.8 °C for the 1 μL water droplet and 38.0 °C for the 5 μL water droplet, much below the thermal degradation temperature of >100 °C for DNA in water under atmosphere pressure^25^. Tracking the internal microfluidic flow, the polystyrene bead solution droplets are imaged at 30 frames per second (Fig. 5C). The polystyrene beads are 3.3 μm in diameter and suspended in water at the concentration of 800 beads/μL. The bead positions were analyzed and overlaid onto the original images using a custom MATLAB program. During a timeframe of 33.3 ms, beads starting at the centric positions (#1, 4, 6, 7) moves toward the apex with a mean velocity of 13.3 mm/s while the edge positions (#2, 3, 5, 8) have a mean velocity of 9.2 mm/s, suggesting strongest flow at droplet center. Overall, polystyrene micro-beads migrate toward the droplet apex at a mean velocity of 11.2 mm/s, in agreement with the proposed coffee-ring reversion flow profile.

## Discussion

To elucidate the experimental results of laser-induced differential evaporation and to support the qualitative arguments how the proposed method can remove the coffee-ring effect, here we present a physical model to connect differential evaporation to coffee-ring effect reversion. Based on the framework of Man and Doi’s analysis on drying pattern^18^, we assume the following two conditions to be approximately satisfied:

1 $${\rm{Droplet}}\,{\rm{height}}\,{\rm{profile}}:h(r,t)=H(t)[1-\frac{{r}^{2}}{{R}^{2}(t)}],$$

2 $${\rm{Overall}}\,{\rm{evaporation}}\,{\rm{rate}}:\dot{V}={\dot{V}}_{o}(\frac{R(t)}{{R}_{o}}).$$

In Eq. (1), the droplet height profile *h*(*r*, *t*) is assumed to follow a parabolic curve described by its center height *H*(*t*) and contact radius *R*(*t*) at time *t*. Eq. (2) describes how the evaporation rate $\dot{V}$ is related to the initial evaporation rate $\dot{V}$_o_ with *R*(*t*) and *R*_*o*_ being the droplet contact radius at time *t* and time 0. Figure 3A,B shows the parabolic curve fit to droplet shape during both laser-induced evaporation and hot plate drying. After initial 30% of drying time progression, parabolic curves fit droplet shapes very well, showing Eq. (1) to be valid for *T*/*T*_0_ > 0.3 (See supplemental Fig. S1). During our laser-heating process, after initial 30% of drying time progression, evaporation rate was proportional to contact radius and independent of contact angle, and thus Eq. (2) was valid for *T*/*T*_0_ > 0.3 (See Fig. S2). The solvent mass conservation equation can be represented as

3 $$\frac{d}{dt}{\int }_{0}^{r}dr\text{'}2\pi r\text{'}h({r}^{\text{'}},t)=-2\pi rv(r,t)h(r,t)-{\int }_{0}^{r}dr\text{'}2\pi r\text{'}J(r\text{'},t),$$

where *v*(*r*, *t*) denotes the height-average fluid velocity and *J*(*r*, *t*) denotes the local solvent evaporation rate per unit area (Vol/s-area). The sign of the velocity is positive for fluid leaving the center and negative towards the center. The evaporation rate, *J*(*r*, *t*) has been shown by previous studies^18^ as

4 $$J(r,t)=-\frac{{\dot{V}}_{o}}{\pi {R}_{o}R(t)}.$$

To describe laser-induced differential evaporation, we have modified *J*(*r*, *t*) in Eq. (4) as $J(r,t)={J}_{i}+{J}_{d}$, where *J*_*i*_ is the evaporation rate due to isothermal evaporation because of laser heating, and *J*_*d*_ is the differential evaporation by the focused laser beam. From the measured temperature profile of the droplet under the focused CO_2_ laser beam and the fact that laser-induced evaporation dries the droplet 10 times faster than the uniformly heated droplet to 50 °C by a hot plate, we can assume that ${J}_{d}\gg {J}_{i}$:

5 $$\begin{array}{c}J(r,t) \sim {J}_{i}\cong 0\,{\rm{if}}\,r > a\\ J(r,t) \sim {J}_{d}=\frac{P}{\pi {a}^{2}}\,{\rm{if}}\,r < a.\end{array}$$

In this expression, we introduced laser-induced evaporation rate *P*(*t*) and laser exposure radius *a*. *P*(*t*) has the unit of Vol/s and is proportional to the droplet surface temperature in the laser irradiated area and to the laser power. Substituting Eq. (5) into Eq. (3) and expanding each individual term, we can obtain the following relation can be obtained:

6 $$2\pi rH(t)v=-\frac{\pi {r}^{2}\dot{H}}{2}[1+\frac{1}{(1-\frac{{r}^{2}}{{R}^{2}})}]+\pi {r}^{2}H\frac{\dot{R}}{R}[1-\frac{1}{(1-\frac{{r}^{2}}{{R}^{2}})}]-\frac{P}{[1-\frac{{r}^{2}}{{R}^{2}}]},$$

where $\dot{H}$ is the time derivative of droplet center height. Utilizing Onsager’s principle^26^, $\frac{\partial (\Phi +\dot{F})}{\partial \dot{R}}=0$, Eqs (1), (2), and (6), and the assumption that the droplet volume decreases much faster than the equilibration of the contact angle (i.e., the ratio between the characteristic time of drying a droplet and that of relaxing to contact angle, ${K}_{ev}\gg 1$, see Eq. S22 for definition of *K*_*ev*_ and Eq. S5 for Onsager’s principle), we can approximate the rate of contact radius movement $\dot{R}$ as

7 $$\dot{R} \sim \frac{R\dot{V}}{4(1+{k}_{cl})V}+\frac{RP}{4V(1+{k}_{cl})}(1+\frac{1}{C}),$$

where *k*_*cl*_ denotes the ratio of contact line friction to hydrodynamic friction and $C=[-ln({\epsilon })-1]$. ${\epsilon }$ is a small number introduced to avoid singularity and is defined as ${\epsilon }={\mathrm{lim}}_{r\to (1-{10}^{-6})R}(1-\frac{{r}^{2}}{{R}^{2}})$. In our analysis, we follow Man and Doi’s assumption that the ratio of contact line friction to hydrodynamics friction, *k*_*cl*_ is a time-independent material parameter that is determined by the droplet and the substrate (see Eq. S19 for mathematical definition of *k*_*cl*_).

To analyze the coffee-ring effect, we need to find where and when the solutes precipitate during the droplet evaporation process. We assume that the solute moves at the same velocity as the fluid inside droplet before precipitation. We define $\tilde{r}({r}_{{\rm{o}}},t)\,$as the height-averaged position of a solute at time t with their initial radial position at *r*_*o*_, and $(\tilde{r})$ as its time derivative representing the speed of movement of the solute as the droplet evaporates. Based on the above assumption for *k*_*cl*_ and from Eq. (7), we obtain the following relation between the solute speed, solute position, and the state of the droplet under laser-induced evaporation:

8 $$\frac{(\tilde{r})}{\tilde{r}}({r}_{o},t)=-{k}_{cl}\frac{\dot{R}}{R}-\frac{P}{2\pi H}[\frac{1}{{\tilde{r}}^{2}}-\frac{1}{{R}^{2}}(1+\frac{1}{C})]\,{\rm{for}}\,\tilde{r} > a,$$

Note that the negative sign in front of the $\frac{\dot{R}}{R}$ term means that the solute with an initial position *r*_*o*_ moves in the opposite direction to the droplet radius without laser induced evaporation (i.e. *P* = *0*). At the time *t*_*d*_ when $\tilde{r}=R$, the solute precipitates at the edge of the droplet, revealing the coffee-ring effect. The greater is the coefficient *k*_*cl*_ that is related to liquid viscosity and contact line friction, the more serious the coffee-ring effect becomes. However, when the laser-induced evaporation is set at an appropriate level (to be determined next), and provided the solute at position *r*_*o*_ initially (*t* = 0) precipitates at *t* = *t*_*d*_ at droplet edge ($\tilde{r}({r}_{o},{t}_{d})=R)$, the velocity of solute $(\tilde{r})({r}_{o},{t}_{d})$ can be in the same direction as $\dot{R}$ but at a higher magnitude, yielding a condition that contradicts the presumption that the solute precipitates at the edge of the droplet. Therefore, when the laser induced differential evaporation rate reaches an appropriate level, solute with its initial position *r*_*o*_ will not precipitate at the edge of the droplet, thus removing the coffee-ring effect. A more detailed quantitative analysis is discussed next.

Since Eq. (8) is a function of the laser-induced evaporation rate *P*(*t*), we can control *P*(*t*) to obtain the desired precipitation pattern of solute. A practical approach is to define a mathematical expression for the precipitation profile and find the required laser induced evaporation rate such that the resulting precipitation profile is bounded by the mathematical expression. From (8), we can easily find that without laser-induced evaporation, the position of the solute is related to the droplet radius as: $\tilde{r}({r}_{o},t)={r}_{o}{(\frac{R}{{R}_{o}})}^{-{k}_{cl}}$, which will lead to the coffee-ring pattern as explained previously. To counter the coffee-ring effect, we introduce a parameter *G* > 0 to alter the relation into (9) and use the laser power to control the value of *G*:

9 $$\tilde{r}({r}_{o},t)={r}_{o}{(\frac{R}{{R}_{o}})}^{-[{k}_{cl}-G]},$$

To overcome the coffee-ring effect, *G* needs to be of sufficient strength such that $G > {k}_{cl}+1.27$ (see Eq. S30 for derivation of the condition for *G* in the supplementary information). From Eqs (8) and (9) and the criterion for *G*, we show that *P*(*t*) needs to satisfy the following criterion:

10 $$P(t) > -GR\dot{R}(2\pi H)=\frac{G\theta }{2}|\frac{d}{dt}(\frac{2\pi {R}^{3}}{3})|.$$

To obtain the last expression of Eq. (10), we have applied the relation that the contact angle $\theta  \sim \frac{2H}{R}$ is time independent for the CCA mode as discussed before. The term $|\frac{d}{dt}(\frac{2\pi {R}^{3}}{3})|$ is the rate of change of the volume of “hypothetical half dome” of the droplet even though the actual shape of the evaporation droplet is not semi-spherical.

The result suggests that by controlling the laser power according to Eq. (10), the solute precipitation behaviors will be within the bound of Eq. (9). During our laser-heating process, laser power was gradually decreased from 40 to 0 mW over the 90 second drying period, as suggested by Eq. (10). Assuming the amount of solute initially present between *r*_*o*_ and *r*_*o*_ + *dr*_*o*_ is later on precipitated between $\tilde{r}$ and $\tilde{r}+d\tilde{r}$, we then have the relation $2\pi {\varphi }_{o}h({r}_{o}){r}_{o}d{r}_{o}=2\pi u(\tilde{r})\tilde{r}d\tilde{r}$, which can be written as

11 $$u(\tilde{r})={\varphi }_{o}h({r}_{o})\frac{{r}_{o}}{\tilde{r}}{(\frac{d\tilde{r}}{d{r}_{o}})}^{-1}.$$

where ${\varphi }_{o}$ is the initial solute concentration and $u(\tilde{r})$ is the drying pattern deposit density.

The solute precipitation condition $\tilde{r}({r}_{o},{t}_{d})=R({t}_{d})$ at time *t*_*d*_, Eq. (9) gives rise to the following relation:

12 $$\tilde{r}({r}_{o},{t}_{d})=R({t}_{d})={{r}_{o}}^{\frac{1}{1+{k}_{cl}-G}}{{R}_{o}}^{\frac{{k}_{cl}-G}{1+{k}_{cl}-G}}.$$

**Figure 6:**
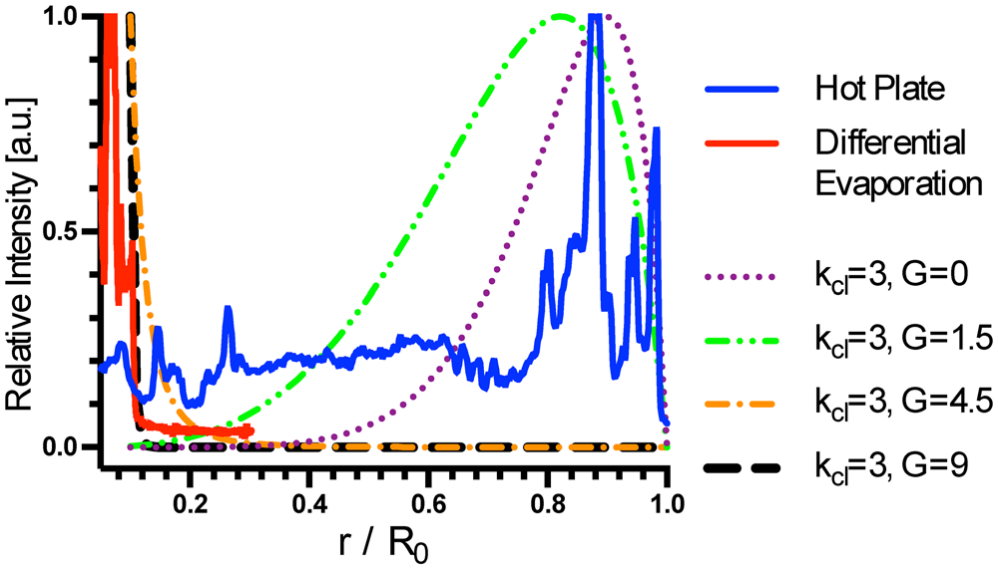
Model fitting of drying pattern analytical solution to drying patterns of laser-induced differential evaporation and hot plate heating. Solute densities are normalized to their respective maximum values. The droplet volume is assumed to decrease much faster than the equilibration of the contact angle (${K}_{ev}\gg 1$, see Eq. S21 for definition of *K*_*ev*_). *k*_*cl*_ is a time-independent material parameter that is determined by the droplet and the substrate. *G* is related to laser-induced evaporation rate *P*(*t*) by Eq. (10). For all cases, material parameter *k*_*cl*_ = 3. For *G* = 0 (purple curve), the analytical solution is the same as in Man and Doi’s analysis and closely matches to the drying pattern of hot plate heating, a characteristic coffee-ring. For *G* = 4.5 (orange curve), the analytical solution closely matches to the drying pattern of laser-induced differential evaporation, a characteristic center peak.

Substituting Eq. (12) into Eq. (11), we derive the drying pattern deposit density

13 $$u(\tilde{r})={\varphi }_{o}{H}_{o}(1+{k}_{cl}-G){(\frac{\tilde{r}}{{R}_{o}})}^{2[{k}_{cl}-G]}[1-{(\frac{\tilde{r}}{{R}_{o}})}^{2(1+{k}_{cl}-G)}],$$

where ${\varphi }_{0}$ is the initial droplet height at *t* = 0. In Fig. 6, $\frac{u(\tilde{r})}{{\varphi }_{o}{H}_{o}}$ is normalized to its maximum value and plotted against $\frac{\tilde{r}}{{R}_{o}}$ to show changes of drying pattern deposit density from center to edge of the initial droplet.

Since DNA concentration was highly enriched from its initial concentration of 10 nM and the heated evaporations (laser and hot plate) occurred much faster than natural drying, we assumed ${K}_{ev}\gg 1$. To fit the drying pattern of hot-plate hitting, the material parameter *k*_*cl*_ = 3 is chosen. The result clearly shows the coffee-ring effect. By increasing the laser-induced evaporation rate to increase the value of *G*, the deposit peak moves toward the droplet center. At *G* = 4.5, the drying pattern shape shifts from coffee-ring to center peak pattern, in agreement with the observed drying pattern of laser-induced differential evaporation. Therefore, the model provides not only a quantitative relation of the effect of laser-induced differential evaporation on the drying pattern, but also experimental guidelines about laser power dependence on the droplet size.

In conclusion, using a low power, scalable CO_2_ laser setup to produce differential evaporation over a droplet, we have overcome the coffee-ring effect without any surfactants or additives. As the droplet dries, the solutes precipitate within a predefined area at the center of the droplet. This method allows enrichment and focused deposition of water-soluble molecules, and has potential to substantially advance the technologies in combinational liquid biopsy analysis, ink-jet printing, and microarray fabrication.

## Methods

### Droplet spotting system setup

Fluorescent DNA molecules were diluted in Milli-Q water and spotted using the integrated syringe pump system. The system consisted of a programmable syringe pump (NE-1000, New Era Pump System) mounted with a 1mL plastic syringe (Tuberculin Syringe, Becton Dickinson). The syringe tip was connected to a #27 gauge, stainless metal tip dispensing needle (I.D. 210 μm) via plastic mount and then extended with a 30 cm segment of Tygon tubing before interfacing with a #27 gauge stainless metal tip removed of its plastic stage. For precise displacement control, stainless metal tip at the end of tubing was fixed onto a probe holder integrated to an XYZ linear stage.

### Cytop microarray fabrication

Cytop microarray was patterned with Cytop polymer (Asahi Glass Co., Japan) on 75 × 50 × 1 mm glass slides (Thermo Fisher Scientific, USA). Before Cytop coating, the glass slide was solvent-cleaned and dried. Cytop polymer type A, containing carboxyl end functional group, was used for coating. 0.05% of (3-aminopropyl)triethoxysilane (Sigma Aldrich, USA) in ethanol/water (95/5) mixture were spin-coated on glass to promote Cytop adhesion. A 4.5 μm thick Cytop polymer layer was formed on glass by spin-coating 9% Cytop type A solution at 800 rpm for 30 s and cured at standard Cytop curing condition. To promote photoresist adhesion, Cytop surface was oxygen plasma treated in a microwave plasma system (PS100, PVA Tepla) at 2.45 GHz frequency, gas flow rate of 120 sccm, and power of 200W for 60 s. Using conventional photolithography method, a 5 μm thick negative tone photoresist NR-9 6000PY (Futurrex, USA) was spin-coated onto the Cytop-coated glass and patterned with 100 μm circular opening to Cytop surface. 100 μm well patterns on photoresist were transferred onto the Cytop coating by oxygen plasma etching (Plasmalab 80 plus system, Oxford Instruments) of exposed Cytop surface. After complete etching of Cytop surface, the remaining photoresist was removed by immersion in resist remover (RR41, Futurrex).

### Deposition pattern imaging and analysis

Fluorescent DNA drying pattern on Cytop-coated microarray devices were imaged using an enclosed inverse fluorescent microscope (BZ-9000, Keyence Corporation) at either 5X or 20X magnification depending on the pattern size. The samples were excited by a mercury lamp through a single-band bandpass filter (472.5/30 nM), and the emission light was filtered by another single-band bandpass filter (520/35 nM). Both pattern size and capture ratio analysis were implemented using the ImageJ software. To estimate for the pattern size, ferret diameter of the best-fitting elliptical shapes to the fluorescent DNA pattern was calculated and used. To calculate the “capture ratio” for any given pattern, the integrated fluorescent intensity within the 100 μm microarray pattern was divided by the total integrated fluorescent intensity of the image.

### Data availability

The datasets generated and/or analysed during the current study are available from the corresponding author on reasonable request.

## Electronic supplementary material

Supplementary Information
