# Supplementary material for: Reversing Coffee-Ring Effect by Laser-Induced Differential Evaporation
Source: Sci Rep. 2018 Feb 16;8:3157. doi: 10.1038/s41598-018-20581-0 (PMC5816656; doi:10.1038/s41598-018-20581-0)
Supplement: Supplementary file 1 — Supplementary Information [file 41598_2018_20581_MOESM1_ESM.pdf]

Reversing Coffee-Ring Effect by Laser-Induced Differential Evaporation

Authors: Tony Minghung Yen, Xin Fu, Tao Wei, Roshan Nayak, Yuesong Shi, Yu-Hwa Lo

Supplementary Information

Laser Beam Characterization. The diffraction limited beam spot, D_{fp} , can be calculated from the equation $D_{fp} = \frac{2\,\lambda}{\pi \times N.A.}$ where λ is the laser wavelength and $N.A.$ is the numerical aperture of an optical setup. In our system $N.A. = \frac{D_{beam}}{2\,f}$, where D_{beam} is the laser beam size entering the final plano-convex lens and f is the focal length. Given our laser wavelength, beam size, and focal length being λ=10.6 μm, D_{beam}=1″, and f=50 mm, the diffraction limit of a focused CO_2 laser beam in our setup is 26.6 μm. Table S1 shows the relationship between the observed beam size and distance from focal point to the final plano-convex lens' holder. Beam size was characterized by measuring the incandescent surface area of a 500 μm thick glass substrate. When focusing the laser onto the glass surface, the surface generated incandescence when a critical energy density was reached. During the laser beam size characterization, the circular coverslip attenuator was removed to allow higher energy density. As the distance between glass surface and lens holder approached 46mm, power required to reach incandescence reduces until a minimal beam size of 29 μm was achieved. Overall, we have established a CO_2 laser system that enables precise control of differential evaporation onto liquid sample surface at a spatial resolution of 29 μm.

Verification of key assumptions. As mentioned in the main text, to leverage Man and Doi's analytical results[1], the following conditions on droplet geometry and evaporation rate have to be satisfied:

$$\text{Droplet height profile: } h(r,t) = H(t)\left[1 - \frac{r^2}{R^2(t)}\right], \quad \text{(S1)}$$

$$\text{Overall evaporation rate: } \dot{V} = \dot{V_o}\left(\frac{R(t)}{R_o}\right). \quad \text{(S2)}$$

In Eq. S1, the droplet height profile $h(r,t)$ follows a parabolic curve described by its center height $H(t)$ and contact radius $R(t)$ at time t. To verify Eq. S1, droplet volume from both

parabolic height profile estimation, and its exact volume described by spherical profile were compared:

Estimated droplet volume from parabolic profile: $V(t) = \frac{\pi}{2}\ H(t)R^2(t)$, (S3-1)

Exact droplet volume from spherical profile: $V(t) = \frac{\pi}{6}\ H(t)[3 * R^2(t) + H(t)^2]$. (S3-2)

In Fig. S1, estimated (Eq. S3-1) and exact droplet volumes (Eq. S3-2) of drying droplets (n=3) during laser-induced differential evaporation were calculated and plotted. While the parabolic and spherical droplet volume differs initially, the two curves converge rapidly after 0.3 T/T_0. Convergence of the two curves indicates agreement between actual droplet geometry and mathematical approximation. Therefore, Eq. S1 applies to our empirical results when $T/T_0 > 0.3$.

In Eq. S2, the overall evaporation rate $\dot{V}$ is a linear function of initial evaporation rate $\dot{V}_o$, $R(t)$, and initial contact radius R_o.[2,3] Eq. S2 is valid when the $\dot{V}$ is proportional to contact radius $R(t)$ and independent of contact angle θ, which can be describe by the following condition:

$$\frac{\dot{R}}{R} > \frac{\dot{\theta}}{\theta}, \qquad \text{(S4)}$$

where $\dot{R}$ is the contact radius time derivative and $\dot{\theta}$ is contact angle time derivative. In Fig. S2, absolute values of $\frac{\dot{R}}{R}$ and $\frac{\dot{\theta}}{\theta}$ of drying droplets (n=3) during laser-induced differential evaporation were calculated and plotted. At $T/T_0 = 0.3$, $\frac{\dot{R}}{R}$ outpaces $\frac{\dot{\theta}}{\theta}$ and continues to increase while $\frac{\dot{\theta}}{\theta}$ continues to decrease. When the condition set forth in Eq. S4 is true, droplet evaporation can be contributed mainly to contact radius change and thus Eq. S2 is also true. Therefore, Eq. S2 applies to our empirical results when $T/T_0 > 0.3$.

Derivation of analytic solution for drying pattern of laser-induced differential evaporation. Given Eqs. S1-2 are valid approximation for our laser-induced differential evaporation process, we follow Man and Doi's analysis in finding $\dot{R}$ through the Onsager principle.[1] When applied to Stokesian hydrodynamics, Onsager principle is equivalent to minimization in energy dissipation, which is defined by the Rayleighian $\Re = \Phi + \dot{F}$, and the Onsager principle[4] here is defined as:

$$\frac{\partial(\Phi+\dot{F})}{\partial\dot{R}} = 0, \quad \text{(S5)}$$

where $\dot{F}$ is the time derivative of the free energy of the system and Φ is the energy dissipation function. Define $\theta_e = \sqrt{\frac{2(\gamma_{LS}+\gamma_{LV}-\gamma_{sV})}{\gamma_{LV}}}$ as the equilibrium contact angle:

$$\dot{F} = \gamma_{LV}\left\{\left[\frac{-16V^2}{\pi R^5} + \pi R{\theta_e}^2\right]\dot{R} + \frac{8V\dot{V}}{\pi R^4}\right\}, \quad \text{(S6)}$$

$$\Phi = \left[\frac{1}{2}\int_0^R dr\, 2\pi r\frac{3\eta}{h}v^2\right] + \pi\zeta_{cl}R\dot{R}^2, \quad \text{(S7)}$$

where γ_{LV} is the liquid-vapor interfacial energy density, v is height averaged fluid velocity (v>0 for outward flow and v<0 for inward flow), and ζ_{cl} is inverse of mobility of the contact line ($\zeta_{cl} \to \infty$ if pinned contact line, $\zeta_{cl} \to 0$ if free moving contact line). Defining the solvent mass conservation equation (sign of the velocity: positive for leaving the center, negative towards the center):

$$\frac{d}{dt}\int_0^r dr'\, 2\pi r' h(r',t) = -2\pi r v(r,t)h(r,t) - \int_0^r dr' 2\pi r' J(r',t) \quad \text{(S9)}$$

The evaporation flux $J(r,t)$ [Vol/s-area] can be represented as the sum of two components:

$$J(r,t) = J_i + J_d,$$

where J_i is the evaporation rate due to isothermal evaporation because of laser heating, and J_d is the differential evaporation by the focused laser beam. From the measured temperature profile of the droplet under the focused CO_2 laser beam and the fact that laser-induced evaporation dries the droplet 10 times faster than the uniformly heated droplet (to 50C) by a hot plate, we can assume that $J_d \gg J_i$ and the evaporation flux can be approximated as

$$J(r,t) \sim J_i \cong 0, \qquad r > a$$

$$J(r,t) \sim J_d = \frac{P}{\pi a^2} \qquad r < a \quad \text{(S10)}$$

where a is the radius of the laser selected area. P is the laser-induced evaporation flux and is proportional to the droplet surface temperature in the laser irradiated area. In our case, we only

consider the case r>a to remove the coffee-ring effect outside the laser spot. In a droplet with r>a, from Eqs. S9-10,

$$\int_0^r dr' 2\pi r' J(r',t) = P. \qquad \text{(S11)}$$

From Eqs. S9 and S11,

$$\frac{d}{dt}\int_0^r dr'\, 2\pi r' h(r',t) = -2\pi r v(r,t) h(r,t) - P. \quad \text{(S12)}$$

Each term in Eq. S12 can be represented as below:

$$\frac{d}{dt}\int_0^r dr'\, 2\pi r' H(t)\left[1-\frac{r'^2}{R^2(t)}\right] = \pi r^2 \dot{H} - \frac{\pi r^4}{2}\frac{d}{dt}\frac{H}{R^2} = \pi r^2 \dot{H} - \frac{\pi r^4}{2}\frac{\dot{H}}{R^2} + \frac{2\pi r^4}{2}\frac{H}{R^3}\dot{R},$$

$$2\pi r v(r,t) h(r,t) = 2\pi r H(t)\left[1-\frac{r^2}{R^2(t)}\right] v.$$

Substitute all the above into Eq. S12 to derive Eq. S13

$$\pi r^2 \dot{H} - \frac{\pi r^4}{2}\frac{\dot{H}}{R^2} + \frac{2\pi r^4}{2}\frac{H}{R^3}\dot{R} = -2\pi r H(t)\left[1-\frac{r^2}{R^2(t)}\right] v - P$$

$$2\pi r H(t)\left[1-\frac{r^2}{R^2(t)}\right] v + P = -\pi r^2 \dot{H} + \frac{\pi r^4}{2}\frac{\dot{H}}{R^2} - \frac{2\pi r^4}{2}\frac{H}{R^3}\dot{R}$$

$$Right - hand - side = -\frac{\pi r^2 \dot{H}}{2}\left[2-\frac{r^2}{R^2}\right] - \pi r^2 H \frac{\dot{R}}{R}\left(\frac{r^2}{R^2}\right)$$

$$2\pi r H(t)\left[1-\frac{r^2}{R^2(t)}\right] v = -\frac{\pi r^2 \dot{H}}{2}\left[2-\frac{r^2}{R^2}\right] - \pi r^2 H \frac{\dot{R}}{R}\left(\frac{r^2}{R^2}\right) - P$$

$$2\pi r H(t) v = -\frac{\pi r^2 \dot{H}}{2}\left[1+\frac{1}{\left(1-\frac{r^2}{R^2}\right)}\right] + \pi r^2 H \frac{\dot{R}}{R}\left[1-\frac{1}{\left(1-\frac{r^2}{R^2}\right)}\right] - \frac{P}{\left[1-\frac{r^2}{R^2}\right]} \qquad \text{(S13)}$$

Use the following relations:

$$V(t) = \frac{\pi}{2}\; H(t) R^2(t),$$

$$\dot{V} = \frac{\pi}{2}\; \dot{H} R^2 + \pi R H \dot{R},$$

$$\frac{\dot{V}}{V} = \frac{\frac{\pi}{2}\ \dot{H}R^2 + \pi RH\dot{R}}{\frac{\pi}{2}HR^2} = \frac{\dot{H}}{H} + \frac{2\dot{R}}{R}; \qquad \frac{\dot{H}}{4H} = \frac{\dot{V}}{4V} - \frac{\dot{R}}{2R}.$$

We can represent v:

$$v = -\frac{r\dot{H}}{4H}\left[1 + \frac{1}{\left(1-\frac{r^2}{R^2}\right)}\right] + \frac{r}{2}\frac{\dot{R}}{R}\left[1 - \frac{1}{\left(1-\frac{r^2}{R^2}\right)}\right] - \frac{P}{2\pi rH\left[1-\frac{r^2}{R^2}\right]},$$

By reorganizing the terms, we obtain

$$v = r\left[\frac{\dot{R}}{R} - \frac{\dot{V}}{4V}\right] - \frac{r}{\left(1-\frac{r^2}{R^2}\right)}\frac{\dot{V}}{4V} - \frac{P}{2\pi r\left[1-\frac{r^2}{R^2}\right]H}, \qquad \text{(S14)}$$

Substitute Eq. S14 into Eq. S7,

$$\Phi = \left[\frac{1}{2}\int_0^R dr\, 2\pi r\,\frac{3\eta}{h}\left\{r\left[\frac{\dot{R}}{R} - \frac{\dot{V}}{4V}\right] - \frac{r}{\left(1-\frac{r^2}{R^2}\right)}\frac{\dot{V}}{4V} - \frac{P}{2\pi r\left[1-\frac{r^2}{R^2}\right]H}\right\}^2\right] + \pi\zeta_{cl}R\dot{R}^2. \qquad \text{(S15)}$$

$$\Phi = \frac{1}{2}\int_0^R dr\, 2\pi r^3\,\frac{3\eta}{h}\left\{\left[\frac{\dot{R}}{R} - \frac{\dot{V}}{4V}\right]^2 - \frac{1}{\left(1-\frac{r^2}{R^2}\right)}\frac{\dot{V}}{2V}\left[\frac{\dot{R}}{R} - \frac{\dot{V}}{4V}\right] + \frac{1}{\left(1-\frac{r^2}{R^2}\right)^2}\left(\frac{\dot{V}}{4V}\right)^2\right\} - \left[\int_0^R dr\,\frac{3\eta}{h}\left\{r\left[\frac{\dot{R}}{R} - \frac{\dot{V}}{4V}\right] - \frac{r}{\left(1-\frac{r^2}{R^2}\right)}\frac{\dot{V}}{4V}\right\}\frac{P}{\left[1-\frac{r^2}{R^2}\right]H}\right] + \left[\frac{1}{2}\int_0^R dr\,\frac{1}{2\pi r}\frac{3\eta P^2}{H^3\left[1-\frac{r^2}{R^2(t)}\right]^3}\right] + \pi\zeta_{cl}R\dot{R}^2 \qquad \text{(S16)}$$

To avoid singularity in energy dissipation at the contact line in S16, we use a molecular cutoff length to be in the order of $10^{-6}R$ and define $\epsilon = \lim_{r\to(1-10^{-6})R}\left(1 - \frac{r^2}{R^2}\right) = 2x10^{-6}$. The first term in Eq. S16 is

$$\frac{1}{2}\int_0^R dr\, 2\pi r^3\,\frac{3\eta}{h}\left\{\left[\frac{\dot{R}}{R} - \frac{\dot{V}}{4V}\right]^2 - \frac{1}{\left(1-\frac{r^2}{R^2}\right)}\frac{\dot{V}}{2V}\left[\frac{\dot{R}}{R} - \frac{\dot{V}}{4V}\right] + \frac{1}{\left(1-\frac{r^2}{R^2}\right)^2}\left(\frac{\dot{V}}{4V}\right)^2\right\} = \frac{3\pi^2\eta R^4}{4V}\left[-ln(\epsilon) - 1\right]\left(\dot{R} - \frac{R\dot{V}}{4V}\right)^2 - \frac{3\pi^2\eta R^4}{4V}\left(\frac{R\dot{V}}{2V}\right)\left(\dot{R} - \frac{R\dot{V}}{4V}\right)\left[\frac{1}{\epsilon} + ln\epsilon - 1\right] + \frac{3\pi^2\eta R^4}{4V}\left(\frac{R\dot{V}}{4V}\right)^2\left[\frac{1}{2\epsilon^2} - \frac{1}{\epsilon} + \frac{1}{2}\right]$$

Also, the second term in Eq. S16 is

$$\left[\int_0^R dr \frac{3\eta}{h}\left\{r\left[\frac{\dot{R}}{R}-\frac{\dot{V}}{4V}\right]-\frac{r}{\left(1-\frac{r^2}{R^2}\right)}\frac{\dot{V}}{4V}\right\}\frac{P}{\left[1-\frac{r^2}{R^2}\right]H}\right]$$

$$=\frac{3\pi^2\eta R^4}{4V}\frac{PR^2}{2V}\left(\frac{\dot{R}}{R}-\frac{\dot{V}}{4V}\right)\left[\frac{1}{\epsilon}-1\right]-\frac{3\pi^2\eta R^4}{4V}\frac{PR^2}{4V}\left(\frac{\dot{V}}{4V}\right)\left[\frac{1}{\epsilon^2}-1\right]$$

The third term in Eq. S16 is not expanded here since it does not have a $\dot{R}$ component and thus will not be contributing to our derivation of Eq. S17. Use Eqs. S5 (Onsager principle), S6, and S16, then

$$\gamma_{LV}\left[\frac{-16V^2}{\pi R^5}+\pi R{\theta_e}^2\right]+\frac{3\pi^2\eta R^4}{2V}\left(\dot{R}-\frac{R\dot{V}}{4V}\right)\left[-ln(\epsilon)-1\right]-\frac{3\pi^2\eta R^4}{4V}\left(\frac{R\dot{V}}{2V}\right)\left[\frac{1}{\epsilon}+ln\epsilon-1\right]-$$
$$\frac{3\pi^2\eta R^4}{4V}\frac{PR}{2V}\left[\frac{1}{\epsilon}-1\right]+2\pi\zeta_{cl}R\dot{R}=0 \qquad \text{(S17)}$$

Using the approximation: $\dot{V}\sim -P$, Eq. S17 can be written as

$$\gamma_{LV}\left[\frac{-16V^2}{\pi R^5}+\pi R{\theta_e}^2\right]+\frac{3\pi^2\eta R^4}{2V}\left(\dot{R}-\frac{R\dot{V}}{4V}\right)\left[-ln(\epsilon)-1\right]+\frac{3\pi^2\eta R^4}{4V}\left(\frac{RP}{2V}\right)\left[ln\epsilon\right]+2\pi\zeta_{cl}R\dot{R}$$
$$=0$$

Define $C=[-ln(\epsilon)-1]\sim 12$

$$\left(1+\frac{4\pi\zeta_{cl}RV}{3\pi^2\eta R^4 C}\right)\dot{R}-\left(\frac{RP}{4V}\right)\left(1+\frac{1}{C}\right)=\frac{R\dot{V}}{4V}+\frac{2V}{3\pi^2\eta C R^4}\gamma_{LV}\left[\frac{16V^2}{\pi R^5}-\pi R{\theta_e}^2\right], \qquad \text{(S18)}$$

Use the relations: $\theta(t)=\frac{2H(t)}{R(t)}$ and Eq. S3-1,

$$\frac{16V^2}{\pi R^5}=\frac{4\pi H^2}{R},\quad \left[\frac{16V^2}{\pi R^5}-\pi R{\theta_e}^2\right]=\frac{4\pi H^2}{R}-\pi R{\theta_e}^2=\pi R(\theta^2-{\theta_e}^2)$$

$$\frac{2V}{3\pi^2\eta C R^4}=\frac{\pi H R^2}{3\pi^2\eta C R^4}=\frac{\pi H}{3\pi^2\eta C R^2}=\frac{\theta}{6\pi\eta C R},$$

thus the 2nd term in the RHS of Eq. S18 can be written as

$$\frac{2V}{3\pi^2\eta C R^4}\gamma_{LV}\left[\frac{16V^2}{\pi R^5}-\pi R{\theta_e}^2\right]=\frac{\theta\gamma_{LV}}{6\eta C}(\theta^2-{\theta_e}^2)$$

Also the term in the LHS of Eq. S18: $\frac{4\pi\zeta_{cl}RV}{3\pi^2\eta R^4 C}$ can be simplified as

$$\frac{4\zeta_{cl}RV}{3\pi\eta R^4 C} = \frac{\zeta_{cl}\theta}{3\eta C} \equiv k_{cl} \qquad \text{(S19)}$$

As mentioned in the main text, k_{cl} denotes the ratio of contact line friction to hydrodynamic friction. Here, we follow Man and Doi's assumption that k_{cl} is a time-independent material parameter that is determined by the droplet and the substrate. We can simplify Eq. S18 to

$$(1+k_{cl})\dot{R} = \frac{R\dot{V}}{4V} + \frac{\gamma_{LV}}{6\eta C}\theta\left(\theta^2 - {\theta_e}^2\right) + \left(\frac{RP}{4V}\right)\left(1+\frac{1}{C}\right) \qquad \text{(S20)}$$

Consider solute at r_o at t=0. As the solvent evaporates, such a solute is convected by the fluid. $\tilde{r}(r_o, t)$ is the height-averaged position of such a solute at t. Ignoring diffusion, solute moves at the same speed as the fluid as long as it is in the droplet (i.e. $\tilde{r}(r_o, t) < R(t)$). Hence, from Eq. S14,

$$(\dot{\tilde{r}})(r_o, t) = v[\tilde{r}(r_o, t), t] = \tilde{r}\left[\frac{\dot{R}}{R} - \frac{\dot{V}}{4V}\right] - \frac{\tilde{r}}{\left(1-\frac{\tilde{r}^2}{R^2}\right)}\frac{\dot{V}}{4V} - \frac{P}{2\pi\tilde{r}\left[1-\frac{\tilde{r}^2}{R^2}\right]H}.$$

Using the approximate relation: $P = -\dot{V}$, the above equation can be written as

$$(\dot{\tilde{r}})(r_o, t) = v[\tilde{r}(r_o, t), t] = \tilde{r}\left[\frac{\dot{R}}{R} - \frac{\dot{V}}{4V}\right] - \frac{P}{2\pi\tilde{r}H} \qquad \text{(S21)}$$

To help simplifying our final expression, we follow Man and Doi[1] to the following time scales and K_{ev}:

$$\tau_{ev} = \frac{|V_o|}{|\dot{V}_o|} = -\frac{V_o}{\dot{V}_o}, \qquad \tau_{re} = \frac{\eta\, V_0^{\frac{1}{3}}}{\gamma_{LV}\theta_e^3}, \quad K_{ev} = \frac{\tau_{re}}{\tau_{ev}}. \qquad \text{(S22)}$$

where τ_{ev} represents the characteristic time for the droplet to dry completely and τ_{re} represents the characteristic time to relax to its equilibrium contact angle. Assume $K_{ev} \gg 1$ for concentrated polymer (i.e. high viscosity due to enrichment) or strong laser enhanced evaporation effect (i.e. $K_{ev} \gg 1$). Eq. S20 can be approximated as

$$\dot{R} \sim \frac{R\dot{V}}{4(1+k_{cl})V} + \frac{RP}{4V(1+k_{cl})}\left(1+\frac{1}{C}\right), \qquad \text{(S23)}$$

$$\frac{\dot{V}}{4V} = (1+k_{cl})\frac{\dot{R}}{R} - \frac{P}{4V}\left(1+\frac{1}{C}\right) \qquad \text{(S24)}$$

From Eqs. S22 and S24, for $\tilde{r} > a$, we can write Eq. S21 as:

$$(\dot{\tilde{r}})(r_o, t) = v[\tilde{r}(r_o, t), t] = -k_{cl}\tilde{r}(r_o, t)\frac{\dot{R}}{R} + \frac{\tilde{r}P}{4V}\left(1 + \frac{1}{C}\right) - \frac{P}{2\pi\tilde{r}H} \quad \text{for } \tilde{r} > a, \quad \text{(S25)}$$

$$\frac{(\dot{\tilde{r}})}{\tilde{r}}(r_o, t) = -k_{cl}\frac{\dot{R}}{R} + \frac{P}{4V}\left(1 + \frac{1}{C}\right) - \frac{P}{2\pi\tilde{r}^2 H},$$

$$\frac{(\dot{\tilde{r}})}{\tilde{r}}(r_o, t) = -k_{cl}\frac{\dot{R}}{R} - \frac{P}{2\pi H}\left[\frac{1}{\tilde{r}^2} - \frac{1}{R^2}(1 + \frac{1}{C})\right], \quad \text{(S26)}$$

Note that because of the negative sign in front of the $\frac{\dot{R}}{R}$ term, the solute moves in the opposite rate as the droplet when the laser induced evaporation rate is turned off, revealing the coffee-ring effect. The greater is the coefficient k_{cl} that is related to liquid viscosity and contact line friction, the more serious the coffee-ring effect becomes. However, when the laser-induced evaporation is set at an appropriate level (to be determined next), and provided the solute at position $r_o$initially ($t = 0$) precipitates at $t = t_d$ at droplet edge ($\tilde{r}(r_o, t_d) = R$), the velocity of solute $(\dot{\tilde{r}})(r_o, t_d)$ can be in the same direction as $\dot{R}$ but at a higher magnitude, yielding a condition that contradicts the presumption that the solute precipitates at the edge of the droplet. Therefore, when the laser induced differential evaporation rate reaches an appropriate level, solute with its initial position r_o will not precipitate at the edge of the droplet, thus removing the coffee-ring effect.

Since Eq. S26 is a function of the laser-induced evaporation rate $P(t)$, we can design $P(t)$ to approximately yield the following solution that will produce the deposition pattern to be discussed next:

$$\tilde{r}(r_o, t) = r_o\left(\frac{R}{R_o}\right)^{-[k_{cl} - G]}, \quad \text{(S27)}$$

with r_o being the initial solute position (at t=0) within the droplet and $\tilde{r}$ being the solute position at time “t”. Inserting Eq. S27 into Eq. S26, we can obtain

$$GR\dot{R} = -\frac{P}{2\pi H}\left[\frac{R^2}{\tilde{r}^2} - (1 + \frac{1}{C})\right],$$

$$\text{since } \frac{R^2}{\tilde{r}^2} = \frac{R^2}{{r_o}^2}\frac{{R_o}^{2[G-k_{cl}]}}{R^{2[G-k_{cl}]}}$$

$$GR\dot{R} = -\frac{P}{2\pi H}\left[\frac{R^2}{{r_o}^2}\frac{{R_o}^{2[G-k_{cl}]}}{R^{2[G-k_{cl}]}} - (1+\frac{1}{C})\right]. \qquad \text{(S28)}$$

By choosing $G \gg k_{cl} + 1$ to remove coffee-ring and keeping in mind that $GR\dot{R} < 0$ since $\dot{R} < 0$, then

$$GR\dot{R} \sim -\frac{P}{2\pi H}\left[\frac{R^2}{{r_o}^2}\frac{R_o^{2[G-k_{cl}]}}{R^{2[G-k_{cl}]}} - \left(1+\frac{1}{C}\right)\right] \sim -\frac{P}{2\pi H}\left[\frac{R^2}{{r_o}^2}\frac{R_o^{2[G-k_{cl}]}}{R^{2[G-k_{cl}]}}\right]. \text{(S29)}$$

To assure that $\frac{R^2}{{r_o}^2}\frac{R_o^{2[G-k_{cl}]}}{R^{2[G-k_{cl}]}} \gg (1+\frac{1}{C})$, we must have $\left(\frac{R_o}{R}\right)^{2(G-k_{cl}-1)} \gg (1+\frac{1}{C})$ and thus we can set a condition for *G* in Eq. S27 as

$$G > k_{cl} + 1 + \frac{\ln 1.08}{2\ln\left(\frac{R_0}{R}\right)}. \qquad \text{(S30)}$$

While $R \to R_0$ leads to $G \to \infty$, please note that our parabolic volume profile is only valid for timeframe $T/T_0 > 0.3$. From Fig. 3, $\ln\frac{R_0}{R} > 0.15\ at\ T/T_0 = 0.4$, and thus $G > k_{cl} + 1.27$. To prevent the solute that is initially located at r_o from precipitation, we set the laser-induced evaporation rate to satisfy the following condition:

$$P(t) > -GR\dot{R}(2\pi H)\left(\frac{R}{R_o}\right)^{2(G-k_{cl}-1)}\left(\frac{r_o^2}{R_o^2}\right)$$

Since $r_o \leq R_o$ and $R \leq R_o$, we can safely set the laser power to be the following to assure that the real solution of Eq. S29 is bounded by the expression of Eq. S27.

$$P(t) > -GR\dot{R}(2\pi H) = GH\frac{d}{dt}(\pi R^2) \qquad \text{(S31)}$$

Assume the solute eventually precipitates at position $\tilde{r}(r_o, t_d) = R(t_d)$, from Eq. S27,

$$\tilde{r}(r_o, t_d) = R(t_d) = (r_o)^{\frac{1}{1+k_{cl}-G}}(R_o)^{\frac{k_{cl}-G}{1+k_{cl}-G}}. \qquad \text{(S32)}$$

Inserting Eq. S32 into the expression of drying pattern deposit density,

$$u(\tilde{r}(r_o, t)) = h(r_o, 0)\emptyset_o\frac{r_o}{\tilde{r}}\left(\frac{d\tilde{r}}{dr_o}\right)^{-1}, \quad \text{(S33)}$$

where $\emptyset_o$ is initial solute concentration (at $t = 0, R = R_o$) and H_o is the initial droplet height at $t = 0$, and representing r_o in terms of $\tilde{r}$, Eq. S33 becomes

$$u(\tilde{r}) = \emptyset_o H_o (1 + k_{cl} - G) \left(\frac{\tilde{r}}{R_o}\right)^{2[k_{cl}-G]} \left[1 - \left(\frac{\tilde{r}}{R_o}\right)^{2(1+k_{cl}-G)}\right], \quad \text{(S34)}$$

which is our final solution for drying pattern of laser-induced differential evaporation.

Supplementary Figures and Tables

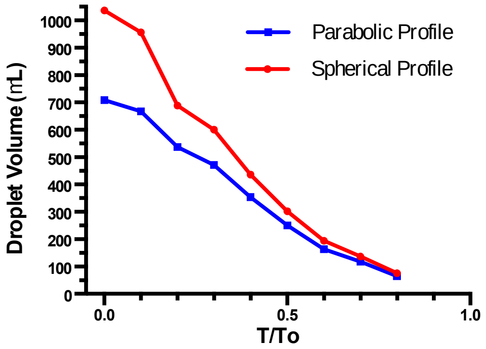

Fig. S1. Estimated droplet volume from parabolic profile vs. exact droplet volume from spherical profile of drying droplets (n=3) during laser-induced differential evaporation.

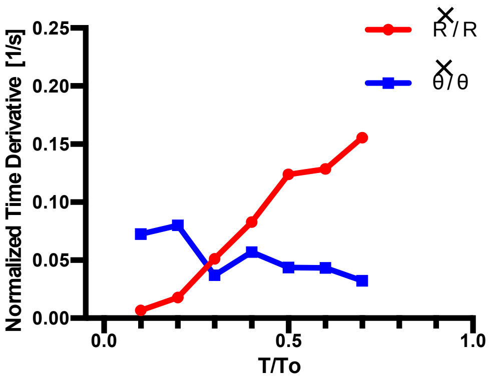

Fig. S2. $\dot{R}/R$ and $\dot{\theta}/\theta$ for of drying droplets (n=3) during laser-induced differential evaporation.

Table S1. Observed Beam Size vs. Distance to Lens

Distance to Lens (mm)	Power (mW)	Beam Size (μm)
45	160	60
45.5	125	48
46	70	29
46.5	104	40
47	181	69

Supplementary Information References

1. Man, X. & Doi, M. Ring to Mountain Transition in Deposition Pattern of Drying Droplets. *Phys. Rev. Lett.* **116,** 1–5 (2016).

2. Kobayashi, M., Makino, M., Okuzono, T. & Doi, M. Interference effects in the drying of polymer droplets on substrate. *J. Phys. Soc. Japan* **79,** 4–9 (2010).

3. Parisse, F. & Allain, C. Drying of Colloidal Suspension Droplets: Experimental Study and Profile Renormalization. *Langmuir* **13,** 3598–3602 (1997).

4. Doi, M. Onsager principle as a tool for approximation. *Chinese Phys. B* **24,** 020505 (2015).
